# The effects of a gamified approach avoidance training and verbal suggestions on food outcomes

**DOI:** 10.1371/journal.pone.0201309

**Published:** 2018-07-26

**Authors:** Lemmy Schakel, Dieuwke S. Veldhuijzen, Henriët van Middendorp, Pieter Van Dessel, Jan De Houwer, Rafael Bidarra, Andrea W. M. Evers

**Affiliations:** 1 Faculty of Social and Behavioural Sciences, Institute of Psychology, Health, Medical and Neuropsychology Unit, Leiden University, Leiden, The Netherlands; 2 Leiden Institute for Brain and Cognition, Leiden University, Leiden, the Netherlands; 3 Department of Experimental Clinical and Health Psychology, Ghent University, Ghent, Belgium; 4 Department of Intelligent Systems, Delft University of Technology, Delft, The Netherlands; 5 Department of Psychiatry, Leiden University Medical Centre, Leiden, The Netherlands; Univerity of Salzburg, AUSTRIA

## Abstract

There is initial support for the effectiveness of approach-avoidance trainings in altering food-related health behaviors. Furthermore, outcome expectancies induced by verbal suggestions might optimize the effectiveness of these interventions, as shown in placebo research. The present study investigated the effectiveness of a gamified approach-avoidance training on food-related outcomes and whether verbal suggestions could strengthen those effects. A total of 120 participants were randomly assigned to 1 of 4 conditions: serious gaming only, verbal suggestions only, serious gaming combined with verbal suggestions, or a gaming control condition. Virtual food preference and food choice were assessed with a food choice task, with pairs differing in healthiness or in healthiness and attractiveness. Implicit food preference was assessed with an Implicit Association Test and food intake with a bogus taste test. Participants in both serious gaming conditions made healthier food choices for pairs differing in healthiness and attractiveness and had healthier implicit food preferences compared to gaming control. No effects were found on food intake. These findings provide the first preliminary support for the effects of a gamified approach-avoidance training on virtual food choice and implicit food preference. Future studies should further elucidate these effects, also in other health domains such as physical activity.

## Introduction

Repeated exposure to appetitive food-related cues can result in approach biases towards unhealthy food products. These biases, in turn, can translate into unhealthy food behaviors [1–5]. Such cognitive biases can be altered by applying approach-avoidance interventions (i.e., repeatedly approaching or avoiding certain stimuli) [6]. There is some initial support for the effectiveness of approach-avoidance interventions in altering food-related stimulus evaluations, as reflected in reduced approach biases towards unhealthy food stimuli [4, 7–9]. The results of approach-avoidance interventions on actual health behaviors such as food consumption are, however, less conclusive. More specifically, one study did find positive effects of an approach-avoidance intervention on actual food consumption [4], whereas several other studies reported non-significant effects on actual food consumption [2, 7, 10]. A possible explanation for these inconclusive results on actual health behaviors comes from a qualitative study showing that a lack of excitement is often experienced in approach-avoidance interventions due to its repetitive nature [11].

Serious gaming can potentially be a useful tool to enhance the engagement of approach-avoidance interventions. Serious gaming is an umbrella term for computer-delivered interventions that provide training and education in an entertaining way [12]. This innovative tool is increasingly applied in healthcare practice [12, 13], and recent studies in the food-related health domain have provided preliminary evidence for its effectiveness in optimizing food-related outcomes, including food intake [14–16]. A meta-analysis on the effects of serious gaming on healthy lifestyle indicated heterogeneous results, however, which can be at least partially due to the fact that serious games often lack evidence-based interventions [13]. A gamified approach-avoidance training has been investigated in one study so far, in the alcohol domain, which showed that a gamified approach-avoidance training produced similar results as a more traditional approach-avoidance training paradigm [17]. Although the effects of non-gamified approach-avoidance training have been investigated before in various domains, including healthy food behavior [2, 4, 7, 10], the effects of gamified approach-avoidance training have not yet been investigated in healthy food behavior.

Besides the lack of excitement that people often experience when completing approach-avoidance interventions, it was shown that those interventions are often faced with a lack of perceived credibility towards the helpfulness of such interventions [11]. Verbal suggestions can possibly optimize the effectiveness of gamified approach-avoidance trainings, since verbal suggestions are able to optimize perceived treatment credibility and health outcomes, as shown previously particularly in placebo research [18, 19]. First, this might be accomplished by influencing expectancies regarding the effectiveness of an intervention, i.e., outcome expectancies [18, 20]. Prior studies have demonstrated the effectiveness of outcome expectancies induced by verbal suggestions in relieving itch and pain in healthy participants [21, 22], and have shown that verbal suggestions are able to induce analgesic effects in various clinical patient populations, including patients with irritable bowel syndrome and patients undergoing thoracotomy [23–25]. A second way to influence health outcomes is by means of verbal suggestions that influence specific actions of approaching and avoiding certain stimuli without actually performing these actions, i.e., stimulus-response contingency instructions [26–29]. Verbal suggestions concerning stimulus-response contingencies were recently shown to alter evaluations of fictitious social groups or meaningless words [27–29]. These findings suggest that the effectiveness of gamified approach-avoidance trainings might be strengthened by verbal suggestions.

The present study aimed to investigate the effects of gamified approach-avoidance training on food-related outcomes and whether verbal suggestions could strengthen those effects. In this study, four conditions were compared: a gaming control condition, a serious gaming only condition, a verbal suggestions only condition, and a combined serious gaming and verbal suggestions condition. Virtual food preference and food choice, as assessed by a food choice task, were the primary study outcomes. Secondary outcomes were implicit food preference, as measured by an Implicit Association Test (IAT), and actual food intake, which was measured by a bogus taste test. It was hypothesized that both serious gaming conditions combined (i.e., with or without verbal suggestions) would show improved food-related outcomes compared to the gaming control condition. It was further explored whether the combined serious gaming and verbal suggestions condition would outperform the serious gaming only condition as well as the verbal suggestions only condition. The role of possible moderating factors such as self-control, self-efficacy and healthy eating goal was also explored [30–34].

## Methods

### Ethics statement

The protocol was approved by the local psychological ethics committee of Leiden University (registration code: CEP16-0728/261) and was preregistered at the Netherlands Trial Register (registration code: NTR6198). The study was performed according to the Declaration of Helsinki (2013).

### Design

The present study used a randomized experimental study design. Participants were randomly allocated, based on a 1:1:1:1 allocation ratio as generated by an online random number generator (www.random.org), to one of the four conditions, stratified for gender. During the experiment, participants were unaware of the existence of four different conditions and therefore blinded for randomization.

### Participants

A total of 120 participants were included in this study. Eligible participants were recruited by written and online flyers which were distributed from September to November 2016 at the campus of Leiden University. Participants had to be fluent in Dutch and between 18 and 35 years old. Exclusion criteria were: (a) severe physical or psychiatric conditions (e.g., chronic somatic diseases affecting daily life or Diagnostic and Statistical Manual of Mental Disorders-Fourth Edition Text Revision [DSM-IV-TR] psychiatric disorders) that interfered with the study protocol, (b) body mass index (BMI) ≥ 30 (given the significant association of obesity with unhealthy lifestyles [35, 36]), and/or (c) having any food restrictions.

### Experimental conditions and control condition

**Fig 1:**
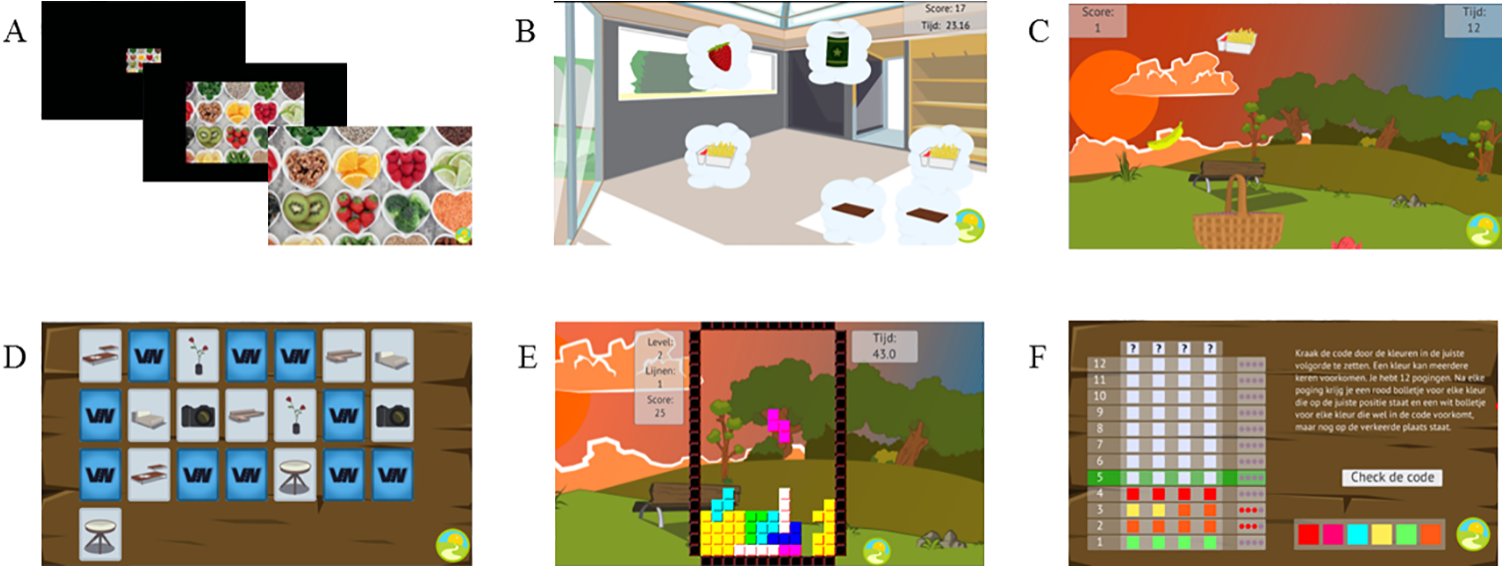
Screenshots of all games. (A) Serious game in which participants were instructed to approach healthy items and avoid unhealthy items by pressing the corresponding arrows on the keyboard; (B) Serious game in which participants were instructed to click away the unhealthy items; (C) Serious game in which participants were instructed to collect the healthy items in a basket; (D) Non-health-related game in which participants were instructed to find and match similar pictures; (E) Non-health-related game in which participants were instructed to complete horizontal lines with various shaped blocks that fell down; (F) Non-health-related game in which participants were instructed to guess a color code by identifying the color pattern.

The serious games and control games were developed in collaboration with Delft University of Technology (ViaNova). See Fig 1 for screenshots of all games.

In all gaming conditions, participants first saw an instruction screen that informed them about the aim of the game. All games were comparable in their appearance and were provided with three different levels of difficulty. All games had a duration around one minute for each game and participants played all three games on the three levels of difficulty twice. In total, participants played 18 games for half an hour, divided into two sessions of 15 minutes, each with a 5-minute break in-between. To motivate participants, they were always rewarded with a virtual medal (golden, silver or bronze) at the end of each game, depending on their performance. In most of the games, participants could earn points and keep track of their performance through a score bar presented at the top of the screen. Accuracy and reaction times were tracked during the games to match the rewards with the performance of participants (note that they were not saved in a log file). In both serious gaming conditions, participants performed a gamified approach-avoidance intervention pertaining to food. Participants were exposed to three different games (see Fig 1A, 1B and 1C) that were all based on the same principle (i.e., approach-avoidance training) aiming to keep participants motivated in playing the games. The food products included in the games entailed various healthy food items, including different types of fruits and vegetables (e.g., pineapple, paprika) and unhealthy food items, including various high-caloric products (e.g., fries, cookies). In all three serious games, participants had to approach healthy items and avoid unhealthy items in an object-referenced way. In two games, participants had to push or click away (avoid) unhealthy items (see Fig 1A and 1B), and in one other game, participants had to focus on healthy items by collecting (approaching) these items and avoiding unhealthy items (see Fig 1C). In the gaming control condition, participants performed three non-health-related computer games, in which the instruction was to match similar non-health-related pictures in one game (see Fig 1D), to complete horizontal lines with different shaped blocks that fell down in another game (see Fig 1E), and to unlock a color code by guessing the color pattern in a third game (see Fig 1F). All games were presented to participants on a computer screen and participants could use the computer mouse and keyboard to play the games.

In the condition that was provided solely with verbal suggestions as well as in the combined serious gaming and verbal suggestions condition, participants received verbal suggestions. The verbal suggestions focused on the effectiveness of the serious games in order to induce outcome expectancies, and also informed participants about stimulus-action contingencies of the approach-avoidance training in the serious games. More specifically, participants were provided with the following verbal suggestions (translated from Dutch):

“*You will play mini games for 15 minutes. After that you will have a break for a few minutes and then you will play the mini games for another 15 minutes*.

*There are three different mini games. In each of these mini games, you will repeatedly respond to healthy and unhealthy stimuli. In the first mini game, you will see healthy and unhealthy food images. Your task is to pull images of healthy food products towards you and to push images of unhealthy food products away. In the second mini game, you will see images of healthy and unhealthy food products flying over. Your task is to keep healthy food products and click unhealthy food products away. In the third mini game, you will learn to make healthy choices. You will do this by catching healthy food products in a picnic basket and avoid the unhealthy food products*.

*Prior research has shown that playing each of these mini games is effective in improving dietary habits*.”

The verbal suggestions were followed by the information that some other tasks first had to be completed before the games would be played (verbal suggestions only condition) or that the games would be played immediately (combined serious gaming and verbal suggestions condition). In the verbal suggestions only condition, it was emphasized to participants that they had to make sure they would not forget the instructions in order to play the games accurately later on. Thereafter, they were exposed to the food-related outcome tasks. After completion of each task, the verbal suggestions regarding the instructions of the games were repeated. Although participants in the verbal suggestions only condition were told that they would play the games after those outcomes, they did not play the games anymore. In the combined serious gaming and verbal suggestion condition, participants were only provided once with the verbal suggestions.

### Food-related outcomes

#### Food choice task

During a computerized food choice task adapted from a previous study [33], participants were presented with seven food product pairs (including one example pair) each containing one healthy food item and one unhealthy food item. As already determined in the previous study of Salmon and colleagues (2014), there were two different types of food product tradeoff pairs of which the first type of product pairs differed in healthiness (i.e., chocolate versus grapes, chocolate cookie versus fruit biscuit, and Dutch caramel waffle versus banana) and the second type of product pairs differed in attractiveness as well by pairing one tasty, unhealthy food product with a healthy, less palatable food product (i.e., chocolate bar versus cereal cookies, crisps versus rice crackers with peanuts, and crisps versus mixed nuts and raisins) [33]. For each pair, participants had to rate how strong their preference was separately for the healthy and unhealthy food product on 7-point scales ranging from 1 (*not at all*) to 7 (*very much*) and they had to indicate which of the two food products they would choose at that moment. A relative food preference was computed for each food product pair by subtracting the unhealthy food preference rating from the healthy food preference rating and subsequently calculating a sum score. Separate scores were determined for the healthiness tradeoff pairs and the healthiness and attractiveness tradeoff pairs. Scores can range from -18 to 18, with higher scores indicating healthier food preferences. Food choice was determined by summing the healthy food choices, with scores ranging from 0 to 3.

#### Implicit association test

The food-related IAT used in the present study was based on a previously validated task [37] with slight changes to some items within categories as to fit the content of the task to the present study purpose. In this task, participants were instructed to categorize pleasant (i.e., happy, smile, peace, joy, pleasure) and unpleasant (i.e., pain, death, poison, sickness, vomit) words, next to healthy (i.e., fruits, banana, vegetables, salad, water) and unhealthy (i.e., chocolate, candy, cake, pastry, cookie) food-related words. The IAT consisted of five blocks. It started with a practice block of ten trials in which food-related words were presented and participants were asked to label these words as either unhealthy (left label) or healthy (right label). Thereafter, another practice block was presented to participants with ten trials in which pleasant and unpleasant words were each presented and participants were asked to assign these words to either positive (left label) or negative (right label) categories. The third and fifth block were test blocks consisting of 40 trials each in which participants had to assign both healthy and unhealthy food-related words, as well as pleasant and unpleasant words to different evaluative categories labeled with ‘unhealthy or positive’ (left label Block 3) or ‘healthy or positive’ (left label Block 5). In the fourth block, consisting of 10 trials, participants again had to categorize food-related words either as healthy or unhealthy, but now with reversed category locations as compared to Block 1 (i.e., left label = ‘healthy’ and right label = ‘unhealthy’). In order to measure the strength of the association between healthy and unhealthy food-related words and the positive and negative valence, participants were instructed to perform the task as fast and accurately as possible. The IAT has been shown be a reliable measure with good predictive validity in measuring behavioral preference towards healthy and unhealthy food items [37]. Implicit food preference was calculated using the D4-algorithm [38], in such a way that higher scores indicate a healthier food preference.

#### Bogus taste test

In order to measure actual food consumption, a bogus taste test was adopted from previous research [39]. Participants were presented with three different unhealthy food products (i.e., 75 grams of crisps, 225 grams of mini Dutch cookies, and 325 grams of M&Ms). These products were presented in separate bowls. For each food product, two identical bowls were presented to participants, who were informed that there were small differences between the food products, whereas these were actually identical. Participants were asked to rate the products from both bowls on various characteristics (e.g., sweetness, crispness) regarding any differences of the food products. Participants were informed that they could eat as much as they wanted and were given 10 minutes to complete their ratings. Unbeknownst to the participants, all bowls were weighed before and after the test in order to explore the total food consumption. Total food consumption was computed by subtracting the weight of all bowls after finishing the taste test from the weight before the start of the taste test.

### Possible moderating factors

#### Self-control

Self-control was measured by the 13-item Brief Self-Control Scale (SCS). Participants completed items on a 5-point scale ranging from 1 (*not at all*) to 5 (*very much*) [34]. Scores on this questionnaire can range from 13 to 65, with higher scores representing higher levels of self-control. The Dutch translation of this questionnaire was used [30], which was found to have a good internal reliability (Cronbach’s alpha = .84).

#### Self-efficacy

The healthy food factor of the Healthy Eating and Weight Self-Efficacy scale (HEWSE) was used to measure self-efficacy. This questionnaire consists of 7 items [40]. Participants completed items on a 5-point scale from 1 (*strongly disagree*) to 5 (*strongly agree*). Scores can range from 7 to 35, with higher scores representing higher levels of self-efficacy. A Dutch translation of the original English version was made by two independent translators applying a forward-backward translation method. A good internal reliability was found in the present study (Cronbach’s alpha = .81), comparable to the original study [40].

#### Healthy eating goal, hunger and appetite

Healthy eating goal was measured by a single item (‘*To what extent do you have the goal to eat healthily*?’) on a 7-point scale ranging from 1 (*not at all*) to 7 (*very much*) [33]. In addition, hunger, appetite and ‘feeling like a bite’ were measured by separate single items (‘*To what extent are you hungry / do you experience appetite / do you feel like a bite at the moment*?’) on a 7-point scale ranging from 1 (*not at all*) to 7 (*very much*).

### Procedure

Prior to participation, participants were informed that the experiment was about games and food, without further detailed information about the actual study purpose, and written informed consent was provided. First, several online questionnaires considering the inclusion and exclusion criteria, demographics, and some other questionnaires not related to the present study aim were completed. If participants were eligible to participate in the study, they were invited for a single lab session that took place at the Faculty of Social and Behavioural Sciences of Leiden University, the Netherlands. Participants were instructed to refrain from eating and drinking except for water for two hours prior to the lab session. At the start of the lab session, baseline psychological characteristics, including self-control, self-efficacy and healthy eating goal, were assessed. After randomization to one of the four conditions, participants were subjected to the food choice task, followed by the IAT and bogus taste test. The order of the IAT and bogus taste test was counterbalanced across participants. In the verbal suggestions condition, the verbal suggestions were repeated after each task. After the tasks, participants had to complete some questionnaires regarding psychological characteristics, which are not described here since they are unrelated to the present study aim. At the end of the session, participants were debriefed about the actual study purpose and received compensation for their participation (€10 or course credits).

### Data preparation and statistical analyses

Data were analyzed using IBM SPSS Statistics for Windows (Version 23; IBM Corporation, Armonk, NY, USA) with a two-tailed significance level of α < .05. The sample size calculation was performed in G*power 3.1 [41]. Based on an effect size *f* of .31 from a previous study on the effects of serious gaming on virtual food preference and food choice [42], a total sample size of 30 participants in each group, including 5 drop-outs (120 in total), was deemed sufficient to obtain a power of .80 with an *α* = .05. Two participants were excluded from the data analyses due to protocol deviations during the lab session (i.e., incorrect sequence of task completion). The data on the covariates were not processed adequately for one participant due to technical problems and for one participant the data on the food choice task were not processed adequately. Furthermore, actual food consumption was not weighed correctly for one participant and one participant did not want to eat one of the food products. Therefore, data of 117 participants were available for analyses on virtual food preference and food choice, as well as for implicit food preference, whereas data of 116 participants were available for analyses on virtual food preference and food choice and data of 115 participants were available for analyses of actual food consumption.

Concerning the food choice task, separate analyses were conducted for pairs differing in healthiness and pairs differing in both healthiness and attractiveness. Because the primary hypothesis of the study was that serious gaming, with or without verbal suggestions, would optimize food outcomes, we performed Analyses of Covariance (ANCOVAs) that tested the effect of the between-subjects factor of Type of Game (serious game vs. control game) by comparing each of the different outcomes for participants in the serious gaming conditions combined, i.e., with and without the verbal suggestions, with food preferences for participants in the gaming control condition. In case there was a significant effect of Type of Game, Holm’s corrected pairwise comparisons were carried out to compare each of the four study conditions (gaming control, serious gaming only, verbal suggestions only, and combined serious gaming and verbal suggestions) separately, in order to receive more insights in the possible effective components of serious gaming (and verbal suggestions). Self-efficacy, self-control, and healthy eating goal were entered as covariates in all analyses.

## Results

### Participant characteristics

151 participants completed the online questionnaire. The eligibility criteria were not met by 26 participants and they were therefore not included in the present study. Five participants did not show up for the lab session. In total, 120 participants (97 women; 80.8%), with an average age of 21.3 years (*SD* = 2.4; range 18–31), completed the study. Baseline characteristics for the four conditions are presented in Table 1. Mean age, gender, BMI, hunger, appetite and feeling like a bite did not differ between the conditions. Also, no significant baseline differences for self-efficacy, self-control, and healthy eating goal were found (all *p*-values > .05).

**Table 1:** Descriptives for the four conditions separately.

	Gaming control (*N* = 28)	Serious gaming (*N* = 29)	Verbal suggestions (*N* = 30)	Serious gaming + verbal suggestions (*N* = 31)
Age	20.89 (1.85)	20.72 (2.42)	22.13 (2.91)	21.32 (2.18)
Body Mass Index	22.53 (2.61)	21.99 (1.78)	22.07 (2.79)	22.21 (2.59)
Sex, *n* female (%)	24 (85.70)	24 (82.80)	23 (76.70)	25 (80.60)
Hunger	3.82 (1.98)	3.97 (1.61)	3.77 (1.85)	4.20 (1.38)^1^
Appetite	4.18 (2.04)	4.66 (1.57)	4.00 (1.74)	4.67 (1.40)^1^
Feeling like a bite	4.36 (2.09)	4.76 (1.41)	4.23 (1.89)	4.97 (1.43)^1^
Self-control (SCS)	38.50 (7.95)	37.21 (9.14)	42.10 (6.61)	39.03 (8.51)
Self-efficacy (HEWSE)	24.07 (5.02)	23.83 (4.72)	24.60 (4.53)	24.70 (5.19)^1^
Healthy eating goal	5.21 (1.07)	4.90 (1.08)	5.10 (0.96)	5.37 (0.85)

^1^*N* = 30.

Note. SCS = Self-Control Scale, HEWSE = Healthy Eating and Weight Self-Efficacy scale

### Virtual food preference

**Fig 2:**
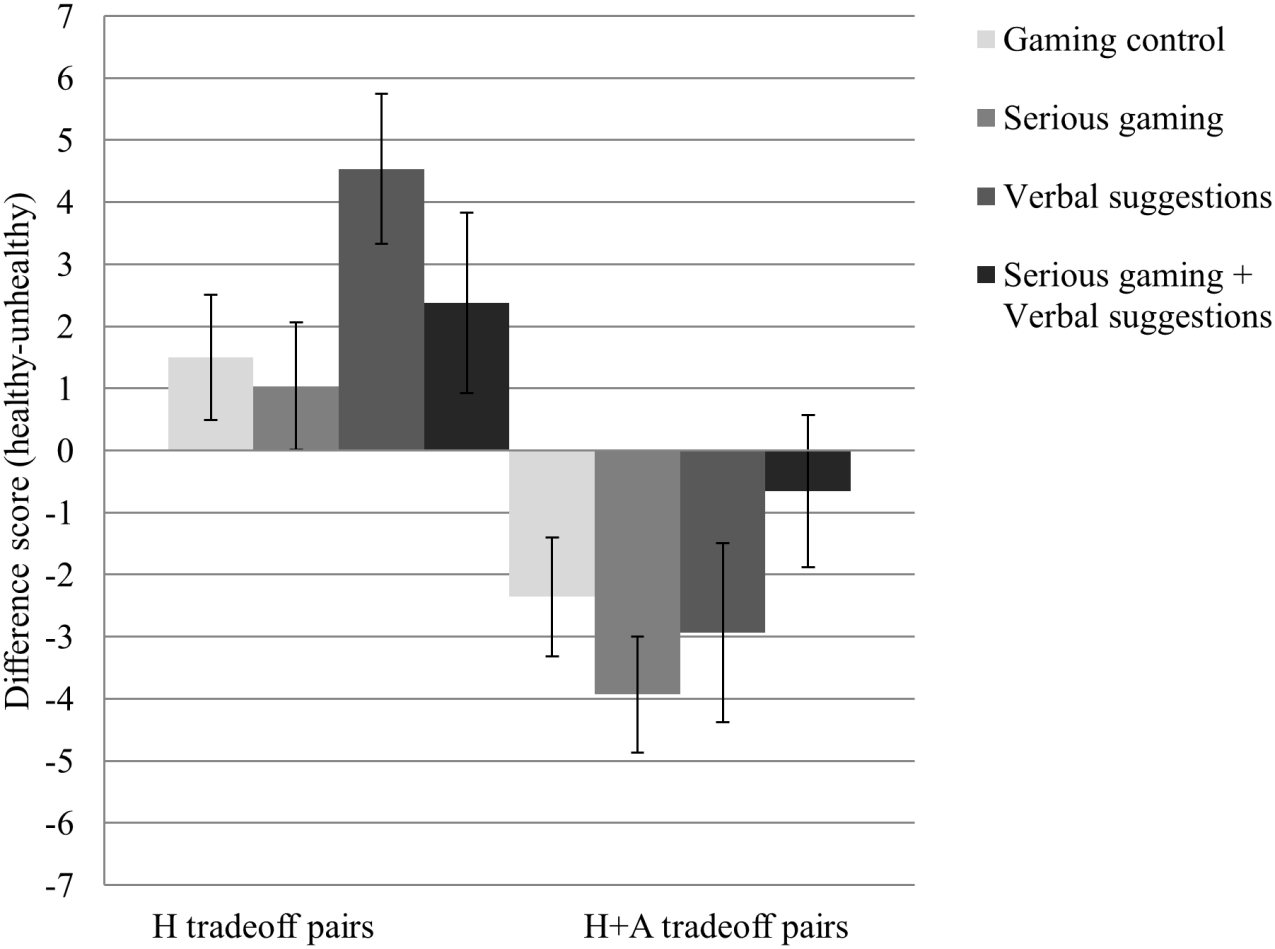
Means and standard errors of the mean for virtual food preference. H tradeoff pairs = Healthiness tradeoff pairs; H+A tradeoff pairs = Healthiness and Attractiveness tradeoff pairs. A higher score on the y-axis represents a more healthy food preference. No significant differences were found in relative food preference between the four conditions on H tradeoff pairs and H+A tradeoff pairs.

The results for food-related outcome measures are presented in Table 2. The virtual food preference for the different conditions is presented in Fig 2. No significant differences between conditions were found for the healthiness tradeoff pairs nor the healthiness and attractiveness tradeoff pairs (both *p-*values > .05).

**Table 2:** Means and standard deviations of food-related outcome measures per condition.

	Gaming control (*N* = 28)	Serious gaming (*N* = 29)	Verbal suggestions (*N* = 30)	Serious gaming + verbal suggestions (*N* = 30)
Food preference H tradeoff	1.50 (5.32)	1.03 (5.52)	4.53 (6.62)	2.38 (7.82)^1^
Food preference H+A tradeoff	-2.36 (5.06)	-3.93 (5.03)	-2.93 (7.89)	-0.66 (6.61)^1^
Food choice H tradeoff	1.89 (0.79)	1.83 (0.89)	2.10 (0.66)	1.90 (1.05)^1^
Food choice H+A tradeoff	0.79 (0.79)	0.97 (0.78)*	1.03 (0.81)	1.34 (0.90)*^1^
Implicit food preference	0.51 (0.64)	0.84 (0.49)*	0.79 (0.50)	0.88 (0.46)*
Food Consumption	58.71 (23.96)	66.04 (26.43)	59.02 (23.18)^2^	66.33 (25.88)

*indicates a significant difference between both serious gaming conditions combined, i.e., with and without the verbal suggestion, and the gaming control condition

^1^*N* = 29.

^2^*N* = 28.

Note. H tradeoff = Healthiness tradeoff, H+A tradeoff = Healthiness and Attractiveness tradeoff

### Virtual food choice

**Fig 3:**
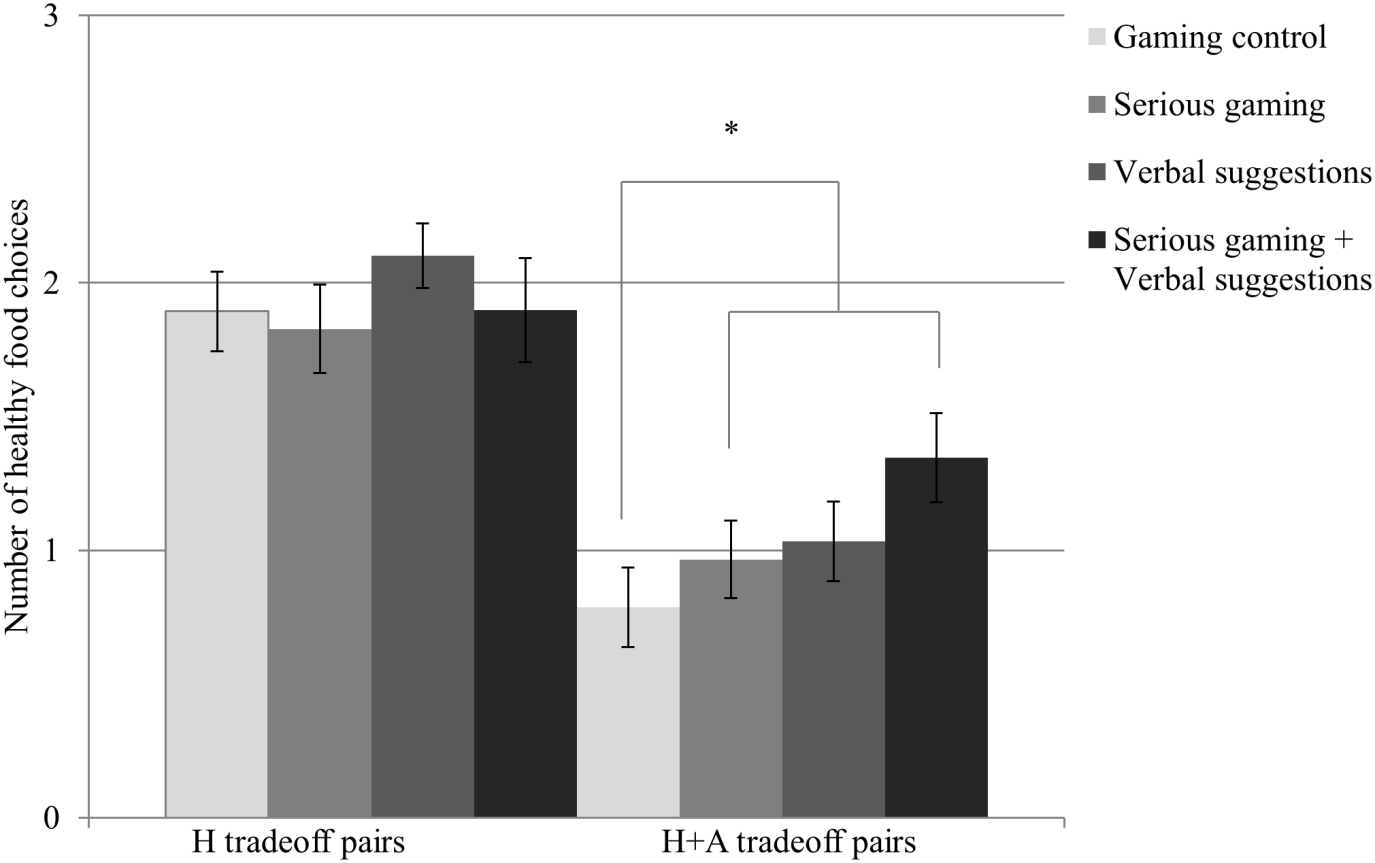
Means and standard errors of the mean for number of virtual healthy food choices. H tradeoff pairs = Healthiness tradeoff pairs; H+A tradeoff pairs = Healthiness and Attractiveness tradeoff pairs. A significant difference was found in that the two serious gaming conditions combined, i.e., with and without the verbal suggestion, showed a higher mean of healthy food choices on H+A tradeoff pairs compared to gaming control. No significant differences between the four conditions were found for H tradeoff pairs.

In Fig 3, virtual food choice for the different conditions is presented. We did not find a significantly healthier virtual food choice on healthiness tradeoff pairs in the serious gaming conditions (*p* = .95).

On pairs differing in healthiness as well as attractiveness, however, a significantly healthier food choice was found for both serious gaming conditions combined, i.e., with and without the verbal suggestions, compared to gaming control, *F* (1,81) = 4.54, *p* = .036, *η*^*2*^ = .13. Pairwise comparisons did not yield any significant differences between the groups (all adjusted *p*-values > .05).

### Implicit food preference

**Fig 4:**
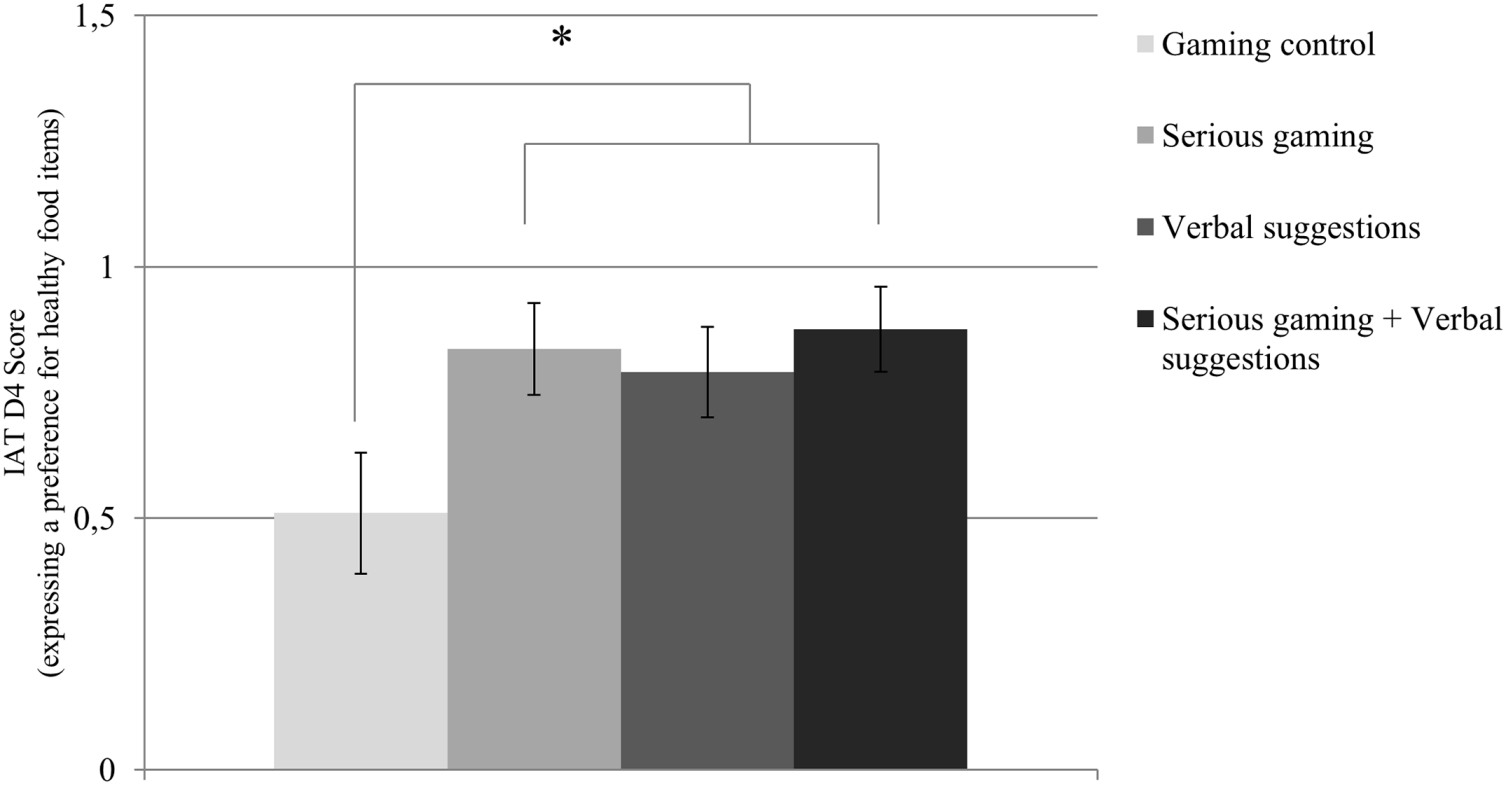
Mean and standard error of the mean for implicit food preference. A higher score on the y-axis represents a more healthy implicit food preference. A significant difference was found in that the two serious gaming conditions combined, i.e., with and without the verbal suggestions, showed a higher implicit preference for healthy food items compared to gaming control.

Implicit food preference outcomes are presented in Fig 4. A significantly healthier implicit food preference was found for both serious gaming conditions combined, i.e., with and without the verbal suggestions, compared to the gaming control condition, *F* (1, 82) = 8.09, *p* = .006, *η*^*2*^ = .14. Pairwise comparisons showed a trend towards a healthier implicit food preference for the combined serious gaming and verbal suggestions condition compared to gaming control, *F* (1, 53) = 6.383, adjusted *p* = .090, *η*^*2*^ = .14.

### Food consumption

**Fig 5:**
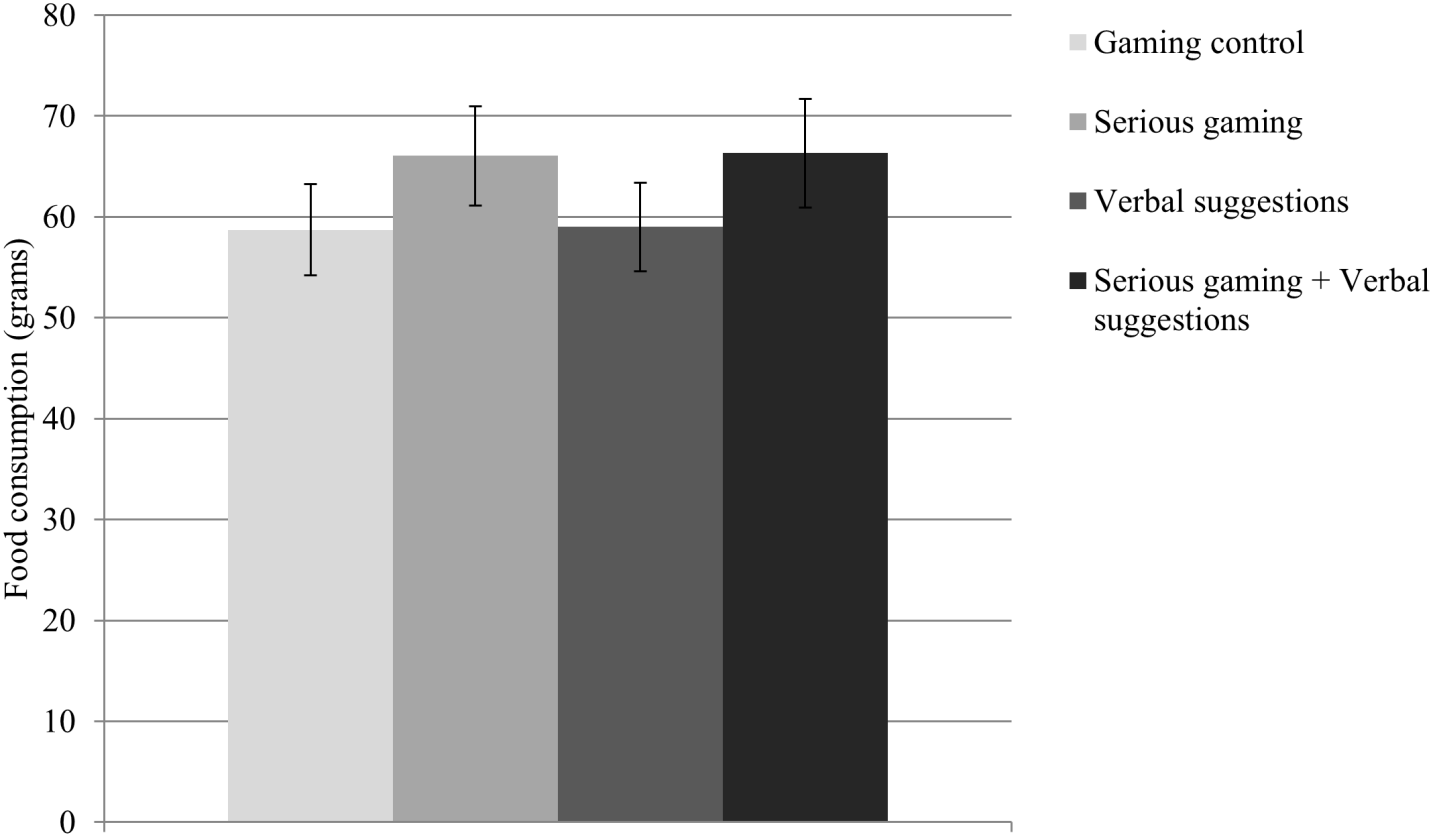
Mean and standard error of the mean for total amount of food consumption. No significant differences between the four conditions were found in total amount of food consumption.

The results for the four conditions on implicit food preference are presented in Fig 5. No significant differences between conditions were found on the bogus taste test (*p* = .17).

## Discussion

The present study used an innovative approach of combining a gamified approach-avoidance training and verbal suggestions to optimize food-related outcomes. A gamified approach-avoidance training resulted in more healthy virtual food choices for pairs differing in both healthiness and attractiveness, and also in a healthier implicit food preference, compared to a non-health-related gaming session. No effects were found on actual food consumption. By investigating the effectiveness of a gamified approach-avoidance training and the add-on effects of verbal suggestions on multiple food-related outcome measures, this study extends current literature on the effectiveness of approach-avoidance trainings in optimizing food outcomes and shows that a gamified approach-avoidance training with or without verbal suggestions affect both virtual and implicit food preference.

Concerning virtual food preference and food choice, the gamified approach-avoidance training resulted in more healthy virtual food choices on pairs differing in healthiness as well as attractiveness compared to playing non-health-related games. On healthiness tradeoff pairs, however, we did not find a healthier virtual food preference and choice after serious gaming. Because the present study included a sample with a relatively high healthy eating goal (i.e., *M* = 5.1 on a 7-point scale), the tradeoff pairs solely differing in healthiness possibly were not challenging enough to optimize these goals even further by means of serious gaming with or without the add-on of verbal suggestions. Instead, opportunities for optimization of food choices are provided by administering a more challenging tradeoff of food pairs differing in healthiness as well as attractiveness, which is intended to generate a self-control conflict [33].

Implicit food preference was also optimized after playing the serious games, in that participants in the serious gaming conditions showed a higher preference for healthy food products over unhealthy food products compared to the gaming control condition. Actual food intake was, however, not affected by playing the serious games and/or providing verbal suggestions. Hence, in our data, the effects of the gamified approach-avoidance training and the add-on of verbal suggestions are restricted to indirect measures of food behaviors, such as virtual food choice and implicit food preference. These discrepant results for direct and indirect measures of food behaviors are partially in line with previous studies on the effects of standard approach-avoidance interventions that also did not find any effects on food consumption [2, 7, 10]. In one study in which participants were trained to approach or avoid chocolate, an effect on food consumption was found in the group that was instructed to avoid chocolate, in that this group ate less of a chocolate muffin, but not of a blueberry muffin, in a subsequent taste test [4]. The results therefore seemed to be restricted to the trained stimulus and did not generalize to other stimuli. Although the present study was not designed to measure possible transfer effects to other food stimuli, future studies should aim to explore possible transfer effects after serious gaming and verbal suggestions by incorporating various food stimuli.

In the present study, the gamified approach-avoidance training was based both on approaching healthy food items and avoiding unhealthy food items. However, the incorporated bogus taste test only consisted of high caloric snack foods, withholding participants from making a healthy food-related choice. Although the majority of bogus taste tests applied in experimental studies are restricted to high caloric snack foods [43], future studies should look into the possibility to develop a more ecologically valid reflection of food consumption, in which healthy food choices can be made as well.

Verbally induced expectancies have been shown to affect health outcomes and combining multiple learning strategies can possibly result in optimized effectiveness of learning processes [18, 44]. For example, the placebo literature demonstrated that strengthening positive expectations towards interventions by verbal suggestions can enhance treatment effectiveness [18, 25, 45]. The verbal suggestions that were incorporated in the present study could have altered participants’ expectations and thereby strengthened the effectiveness of those games. In line with this reasoning the present study found a trend on the IAT indicating more positive implicit food preference for the combined condition compared to the gaming control condition. This trend was not found for the serious gaming only condition or the verbal suggestions only condition, providing some (very preliminary) evidence that playing serious games accompanied by verbal suggestions might be more effective than only playing serious games. Future research should further investigate the added effectiveness of incorporating verbal suggestions in serious gaming.

Interestingly, the results of the verbal suggestions only condition on food-related outcomes seemed comparable with those of the serious gaming only condition and the combined serious gaming and verbal suggestions condition (see Table 2). The fact that the serious games in the present study were primarily based on approach-avoidance training does not mean that effects were the result of low-level mental processes such as the automatic formation of stimulus-response associations on the basis of repeated pairings of stimuli and responses. In accordance with an inferential account of approach-avoidance training effects [46], the training (or even the instructions about stimulus-response contingencies before the training) could also have allowed participants to make inferences about the foods or about the outcomes of food-related actions (e.g., expectancies of positive effects of eating healthy). This could also, at least partially, provide an explanation for the similar effectiveness of verbal suggestions and serious gaming conditions, as solely providing participants with verbal suggestions concerning the actions of serious gaming might be sufficient to make similar inferences that influence food-related outcomes. This observation is in line with previous literature, which already demonstrated that verbal suggestions can change stimulus evaluations not only by repeatedly performing approach and avoidance actions, but also by providing people with verbal suggestions concerning these actions [27–29]. In the present study, however, no significant effects were found for the verbal suggestions only condition. It would nevertheless be interesting to further investigate the effects of verbal suggestions in future research with a specific aim to address the efficacy of verbal suggestions only in optimizing food outcomes which was not a predefined aim of the present study.

Next to the innovative approaches used in the present study to examine the combined effect of a gamified approach-avoidance training and verbal suggestions by incorporating multiple food-related outcome measures, there are several limitations that should be noted as well. First of all, recruitment of a predominantly highly educated student population who had rather high healthy eating goals precludes generalization of findings to a broader population. It is recommended that future studies incorporate other (target) populations in order to further examine the effectiveness of serious gaming and verbal suggestions in optimizing health behaviors. Second, one of the factors contributing to the effectiveness of serious gaming is enhanced engagement [47]. In this study, serious gaming was used in order to enhance engagement of approach-avoidance interventions; however, formal testing of engagement was not included due to the fact that formal indices of engagement are generally lacking. Future studies should aim to develop and incorporate assessments of engagement. Third, although we do not have indications that there was a lack of perceived credibility concerning the provided verbal suggestions, future studies should incorporate a manipulation check (as this might be an important determinant of the effects of verbal suggestions). Additionally, future studies might include a measure of demand compliance because this might provide an explanation of our study results. Note, however, that this explanation does not fit well with the observation that most of the participants were surprised by hearing the study aim. Fourth, previous research provided support for the moderating effects of contingency awareness on implicit and explicit stimulus evaluations [27, 48]. In future research, it would therefore be interesting to evaluate whether contingency awareness moderates the effects of gamified approach-avoidance training and verbal suggestions on food-related outcomes. Fifth, although we minimized the information concerning the actual study aim of the experiment prior to participation, it cannot be ruled out that participants’ responses on the tasks may have been influenced by informing them beforehand that this experiment was about games and food. Sixth, the present study was designed to investigate the combined effects of the serious gaming conditions, i.e., with and without the verbal suggestion, compared to the gaming control condition. In order to draw conclusions on the effectiveness of the individual conditions, future studies should include a larger sample size. Finally, the present study only investigated the effects of serious gaming and verbal suggestions directly after the manipulations. Since we do not know whether those effects will be maintained on a longer term, this should be investigated in future studies by including follow-up assessments. In addition, future studies should evaluate important moderators of serious gaming effects by incorporating various durations of the games and amount of sessions.

The present study was the first in investigating the combined effectiveness of serious gaming and verbal suggestions on food-related outcomes and as such integrates the serious gaming research with research on placebo effects. Some initial support was provided for the effects of serious gaming on virtual food choice and implicit food preference, and possibly the add-on effectiveness of verbal suggestions. By combining innovative ways to alter approach and avoidance tendencies, the present study advances scientific knowledge on the effectiveness of approach-avoidance trainings in optimizing food-related outcomes. Future research should further investigate the effectiveness of serious gaming and the role of verbal suggestions in optimizing food-related outcomes in various target populations and other health domains, such as physical activity.

## Supporting information

S1 Text CONSORT checklist. (DOC)

S2 Text CONSORT flow diagram. (DOC)

## References

[1] Cohen D, Farley TA. Eating as an automatic behavior. Prev Chronic Dis. 2008;5: A23 10.3410/f.1101169.557167 18082012 PMC2248777

[2] Kakoschke N, Kemps E, Tiggemann M. The effect of combined avoidance and control training on implicit food evaluation and choice. J Behav Ther Exp Psychiatry. 2017;55: 99–105. 10.1016/j.jbtep.2017.01.002 28095331

[3] Marteau TM, Hollands GJ, Fletcher PC. Changing human behavior to prevent disease: the importance of targeting automatic processes. Science. 2012;337: 1492–5. 10.1126/science.1226918 22997327

[4] Schumacher SE, Kemps E, Tiggemann M. Bias modification training can alter approach bias and chocolate consumption. Appetite. 2016;96: 219–24. 10.1016/j.appet.2015.09.014 26375357

[5] Havermans RC. Pavlovian craving and overeating: a conditioned incentive model. Curr Obes Rep. 2013;2: 165–70. 10.1007/s13679-013-0053-z

[6] Cristea IA, Kok RN, Cuijpers P. Efficacy of cognitive bias modification interventions in anxiety and depression: meta-analysis. Br J Psychiatry. 2015;206: 7–16. 10.1192/bjp.bp.114.146761 25561486

[7] Dickson H, Kavanagh DJ, MacLeod C. The pulling power of chocolate: Effects of approach–avoidance training on approach bias and consumption. Appetite. 2016;99: 46–51. 10.1016/j.appet.2015.12.026 26725150

[8] Hollands GJ, Prestwich A, Marteau TM. Using aversive images to enhance healthy food choices and implicit attitudes: An experimental test of evaluative conditioning. Health Psychol. 2011;30: 195–203. 10.1037/a0022261 21401253

[9] Kakoschke N, Kemps E, Tiggemann M. Attentional bias modification encourages healthy eating. Eat Behav. 2014;15: 120–4. 10.1016/j.eatbeh.2013.11.001 24411764

[10] Becker D, Jostmann NB, Wiers RW, Holland RW. Approach avoidance training in the eating domain: testing the effectiveness across three single session studies. Appetite. 2015;85: 58–65. 10.1016/j.appet.2014.11.017 25447011

[11] Beard C, Weisberg RB, Primack J. Socially anxious primary care patients’ attitudes toward cognitive bias modification (CBM): a qualitative study. Behav Cogn Psychother. 2012;40: 618–33. 10.1017/S1352465811000671 22127022 PMC3816754

[12] Kato PM. Video games in health care: Closing the gap. Rev Gen Psychol. 2010;14: 113–121. 10.1037/a0019441

[13] DeSmet A, Van Ryckeghem D, Compernolle S, Baranowski T, Thompson D, Crombez G, et al A meta-analysis of serious digital games for healthy lifestyle promotion. Prev Med. 2014;69: 95–107. 10.1016/j.ypmed.2014.08.026 25172024 PMC4403732

[14] Dassen FCM, Houben K, Van Breukelen GJP, Jansen A. Gamified working memory training in overweight individuals reduces food intake but not body weight. Appetite. 2017; 10.1016/j.appet.2017.05.009 28479405

[15] Blackburne T, Rodriguez A, Johnstone SJ. A Serious Game to Increase Healthy Food Consumption in Overweight or Obese Adults: Randomized Controlled Trial. JMIR Serious Games. 2016;13: e10 10.2196/games.5708 27417192 PMC4963607

[16] Shiyko M, Hallinan S, Seif El-Nasr M, Subramanian S, Castaneda-Sceppa C. Effects of Playing a Serious Computer Game on Body Mass Index and Nutrition Knowledge in Women. JMIR Serious Games. 2016;4: e8 10.2196/games.4977 27255497 PMC4911511

[17] Boendermaker WJ, Maceiras SS, Boffo M, Wiers RW. Attentional bias modification with serious game elements: evaluating the shots game. JMIR Serious Games. 2016;4: e20 10.2196/games.6464 27923780 PMC5174726

[18] Colloca L, Miller FG. How placebo responses are formed: a learning perspective. Philos Trans R Soc Lond B Biol Sci. 2011;366: 1859–69. 10.1098/rstb.2010.0398 21576143 PMC3130403

[19] Spagnolo PA, Colloca L, Heilig M. The role of expectation in the therapeutic outcomes of alcohol and drug addiction treatments. Alcohol Alcohol. 2015;50: 282–5. 10.1093/alcalc/agv015 25761920 PMC4415067

[20] Kirsch I. Response expectancy as a determinant of experience and behavior. Am Psychol. 1985;40: 1189–1202. 10.1037/0003-066X.40.11.1189

[21] Peerdeman KJ, van Laarhoven AIM, Bartels DJP, Peters ML, Evers AWM. Placebo-like analgesia via response imagery. Eur J Pain. 2017;21: 1366–1377. 10.1002/ejp.1035 28421648 PMC5573948

[22] Bartels DJ, van Laarhoven AI, Haverkamp EA, Wilder-Smith OH, Donders AR, van Middendorp H, et al Role of conditioning and verbal suggestion in placebo and nocebo effects on itch. PloS One. 2014;9: e91727 10.1371/journal.pone.0091727 24646924 PMC3960153

[23] Vase L, Robinson ME, Verne GN, Price DD. Increased placebo analgesia over time in irritable bowel syndrome (IBS) patients is associated with desire and expectation but not endogenous opioid mechanisms. Pain. 2005;115: 338–47. 10.1016/j.pain.2005.03.014 15911161

[24] Pollo A, Amanzio M, Arslanian A, Casadio C, Maggi G, Benedetti F. Response expectancies in placebo analgesia and their clinical relevance. Pain. 2001;93: 77–84. 10.1016/S0304-3959(01)00296-2 11406341

[25] Peerdeman KJ, van Laarhoven AI, Keij SM, Vase L, Rovers MM, Peters ML, et al Relieving patients’ pain with expectation interventions: a meta-analysis. Pain. 2016;157: 1179–91. 10.1097/j.pain.0000000000000540 26945235

[26] Cohen-Kdoshay O, Meiran N. The representation of instructions operates like a prepared reflex: Flanker compatibility effects found in first trial following S–R instructions. Exp Psychol. 2009;56: 128–33. 10.1027/1618-3169.56.2.128 19261588

[27] Van Dessel P, De Houwer J, Gast A, Smith CT. Instruction-Based Approach-Avoidance Effects. Exp Psychol. 2015;62: 161–169. 10.1027/1618-3169/a000282 25516008

[28] Van Dessel P, De Houwer J, Smith CT. Relational information moderates approach-avoidance instruction effects on implicit evaluation. Acta Psychol. 2017; 10.1016/j.actpsy.2017.03.016 28433196

[29] Van Dessel P, Gawronski B, Smith CT, De Houwer J. Mechanisms underlying approach-avoidance instruction effects on implicit evaluation: Results of a preregistered adversarial collaboration. J Exp Soc Psychol. 2017;69: 23–32. 10.1016/j.jesp.2016.10.004

[30] Adriaanse MA, Kroese FM, Gillebaart M, De Ridder DT. Effortless inhibition: Habit mediates the relation between self-control and unhealthy snack consumption. Front Psychol. 2014;5: 444 10.3389/fpsyg.2014.00444 24904463 PMC4032877

[31] Horwath CC, Nigg CR, Motl RW, Wong KT, Dishman RK. Investigating fruit and vegetable consumption using the transtheoretical model. Am J Health Promot. 2010;24: 324–33. 10.4278/ajhp.071218138 20465146

[32] O’donnell S, Greene GW, Blissmer B. The effect of goal setting on fruit and vegetable consumption and physical activity level in a Web-based intervention. J Nutr Educ Behav. 2014;46: 570–5. 10.1016/j.jneb.2014.03.005 24857600

[33] Salmon SJ, Fennis BM, de Ridder DT, Adriaanse MA, De Vet E. Health on impulse: when low self-control promotes healthy food choices. Health Psychol. 2014;33: 103–9. 10.1037/a0031785 23477580

[34] Tangney JP, Baumeister RF, Boone AL. High self-control predicts good adjustment, less pathology, better grades, and interpersonal success. J Pers. 2004;72: 271–324. 10.1111/j.0022-3506.2004.00263.x 15016066

[35] Drenowatz C. Reciprocal compensation to changes in dietary intake and energy expenditure within the concept of energy balance. Adv Nutr. 2015;6: 592–9. 10.3945/an.115.008615 26374181 PMC4561833

[36] Pearson ES. Goal setting as a health behavior change strategy in overweight and obese adults: a systematic literature review examining intervention components. Patient Educ Couns. 2012;87: 32–42. 10.1016/j.pec.2011.07.018 21852063

[37] Richetin J, Perugini M, Prestwich A, O’Gorman R. The IAT as a predictor of food choice: The case of fruits versus snacks. Int J Psychol. 2007;42: 166–73. 10.1080/00207590601067078

[38] Greenwald AG, Nosek BA, Banaji MR. Understanding and using the implicit association test: I. An improved scoring algorithm. J Pers Soc Psychol. 2003;85: 197–216. 10.1037/0022-3514.85.2.197 12916565

[39] van den Akker K, Schyns G, Jansen A. Enhancing inhibitory learning to reduce overeating: Design and rationale of a cue exposure therapy trial in overweight and obese women. Contemp Clin Trials. 2016;49: 85–91. 10.1016/j.cct.2016.06.008 27353315

[40] Wilson-Barlow L, Hollins TR, Clopton JR. Construction and validation of the healthy eating and weight self-efficacy (HEWSE) scale. Eat Behav. 2014;15: 490–2. 10.1016/j.eatbeh.2014.06.004 25064304

[41] Faul F, Erdfelder E, Buchner A, Lang AG. Statistical power analyses using G*Power 3.1: tests for correlation and regression analyses. Behavior Res Methods. 2009;41: 1149–60. 10.3758/brm.41.4.1149 19897823

[42] Schakel L, Veldhuijzen DS, Manai M, van Beugen S, van der Vaart R, van Middendorp H, et al. Optimizing healthy food preferences by serious gaming. Manuscript submitted for publication. 10.1080/08870446.2019.1675657 31607172

[43] Robinson E, Haynes A, Hardman CA, Kemps E, Higgs S, Jones A. The bogus taste test: Validity as a measure of laboratory food intake. Appetite. 2017;116: 223–31. 10.1016/j.appet.2017.05.002 28476629 PMC5504774

[44] Peerdeman KJ, van Laarhoven AI, Peters ML, Evers AW. An integrative review of the influence of expectancies on pain. Front Psychol. 2016;7: 1270 10.3389/fpsyg.2016.01270 27602013 PMC4993782

[45] Colloca L, Jonas WB, Killen J, Miller FG, Shurtleff D. Reevaluating the placebo effect in medical practice. Z Psychol. 2014;222: 124–7. 10.1027/2151-2604/a000177 25360397 PMC4211281

[46] Van Dessel P, Hughes S, De Houwer J. How Do Actions Influence Attitudes? An Inferential Account of the Impact of Action Performance on Stimulus Evaluation. Manuscript Under Review. https://osf.io/kb3wq/. 10.1177/1088868318795730 30229697

[47] Sardi L, Idri A, Fernandez-Aleman JL. A systematic review of gamification in e-Health. J Biomed Inform. 2017;71: 31–48. 10.1016/j.jbi.2017.05.011 28536062

[48] Van Dessel P, De Houwer J, Gast A. Approach–Avoidance Training Effects Are Moderated by Awareness of Stimulus–Action Contingencies. Pers Soc Psychol Bull. 2016;42: 81–93. 10.1177/0146167215615335 26567171

