# Supplementary material for: The effects of a gamified approach avoidance training and verbal suggestions on food outcomes
Source: PLoS One. 2018 Jul 26;13(7):e0201309. doi: 10.1371/journal.pone.0201309 (PMC6062074; doi:10.1371/journal.pone.0201309)
Supplement: S2 Text — (DOC) [file pone.0201309.s002.doc]

**
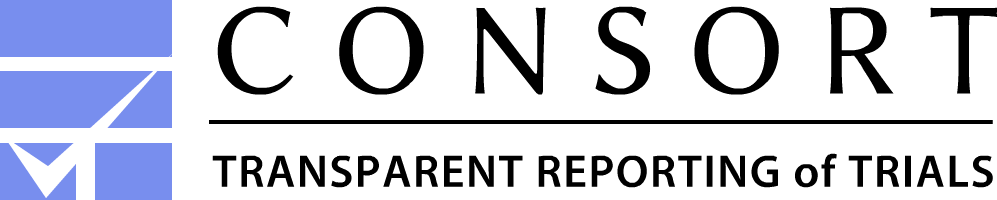
**

**CONSORT 2010 Flow Diagram**

**Allocation**

**Analysis**

**Enrollment**

Assessed for eligibility (n = 151)

Excluded (n = 31)

- Not meeting inclusion criteria (n = 26)
- Did not show up for lab session (n = 5)

Analysed (n= )
 Excluded from analysis (give reasons) (n= )

•Allocated to 1. Gaming control (n = 29)

•Received allocated intervention (n = 29).

•Did not receive allocated intervention (n = 0).

•Allocated 4. Serious gaming and Verbal suggestions (n = 31)

•Received allocated intervention (n = 31).

•Did not receive allocated intervention (n = 0).

Randomized (n = 120)

•Allocated to 2. Serious gaming (n = 30)

•Received allocated intervention (n = 30). •Did not receive allocated intervention (n = 0).

•Allocated to 3. Verbal suggestions (n = 30)

•Received allocated intervention (n = 30).

•Did not receive allocated intervention (n = 0).

•Analysed for implicit association test and bogus taste test (n = 30) and for food choice task (n = 29) •Excluded from all analysis due to technical problems processing covariates (n = 1). •Excluded from analyses of food choice task due to technical problems (n = 1).

•Analysed for food choice task and implicit association test (n = 30) and for food choice task (n = 28) •Excluded from analysis of bogus taste test due to incorrect weighing of the food products (n = 1) and not wanting to eat one of the food products (n = 1).

•Analysed (n = 29)

•Excluded from analysis due to protocol deviations (n = 1).

•Analysed (n = 28)

•Excluded from analysis due to protocol deviations (n = 1).
